# An Aggressive Case of Adrenocortical Carcinoma Complicated by Paraneoplastic Cushing’s Syndrome

**DOI:** 10.7759/cureus.34099

**Published:** 2023-01-23

**Authors:** Mohamed Zakee Mohamed Jiffry, Breeana Hernandez, Meagan Josephs, Aimal Khan, Zaamar Malik

**Affiliations:** 1 Internal Medicine, Danbury Hospital, Danbury, USA; 2 School of Medicine, American University of the Caribbean, Cupecoy, SXM; 3 Psychiatry, Danbury Hospital, Danbury, USA

**Keywords:** chemo radiotherapy (chemo-rt), ectopic cushing’s syndrome, paraneoplastic syndromes, adrenal malignancy, adrenocortical carcinoma (acc)

## Abstract

Adrenocortical carcinoma (ACC) is a rare endocrine malignancy with a poor prognosis. Surgical resection may be curative if localized disease is identified, although recurrence is common. Research shows that the use of adjuvant therapeutic regimens such as EDP-M (combination of mitotane, etoposide, doxorubicin, and cisplatin) in high-risk patients has survival benefits.

A 75-year-old female was incidentally found to have a right adrenal heterogeneous internal enhancement measuring 5.0 x 3.7cm. The workup confirmed autonomous adrenal production of corticosteroids and she was referred to surgery for an adrenalectomy. A T2 ACC with positive margins and lympho-vascular invasion was resected, following which she was started on external beam radiation followed by four cycles of carboplatin and etoposide. Despite initial treatments, she was diagnosed with refractory metastatic disease at subsequent follow-ups. Pembrolizumab immunotherapy was started, but disease progression continued, and she was eventually transitioned to mitotane 1g twice daily. She continued to worsen and was eventually transitioned to hospice care.

The management of ACC remains diagnostically challenging, especially because most patients do not present until an advanced stage of disease. Surgery is commonly employed with a curative intent, and opinions regarding adjuvant cytotoxic therapy and/or radiotherapy remain mixed.

## Introduction

Adrenocortical carcinoma (ACC) is a rare and aggressive endocrine malignancy with an annual incidence of 0.5-2.0 cases per million persons [1]. ACC is associated with an unsatisfactory prognosis with an estimated median survival of about three to four years. The five-year survival is 60-80% for tumors confined to the adrenal space, 35-50% for locally advanced disease, and 0% to 28% in cases of metastatic disease [2].

Surgical en-bloc resection is commonly employed and is recommended for locoregional disease. There is no standard of care for the management of ACC although cytotoxic cisplatin-based regimens such as EDP-M (a combination of mitotane, etoposide, doxorubicin, and cisplatin) may be employed as adjuvant therapy in those with very high recurrence risk. Mitotane is recommended for patients with a high risk of recurrence (stage III disease, R1 resection margins, or Ki67 >10%) although its routine use for low/moderate risk disease is controversial [3]. Despite complete resection of early-stage disease, recurrence rates in ACC are still very high and appropriate management remains a challenge.

We demonstrate a patient with a limited-stage T2 ACC who, despite receiving primary surgery, adjuvant chemotherapy and radiotherapy, progressed to metastatic disease.

## Case presentation

**Figure 1:**
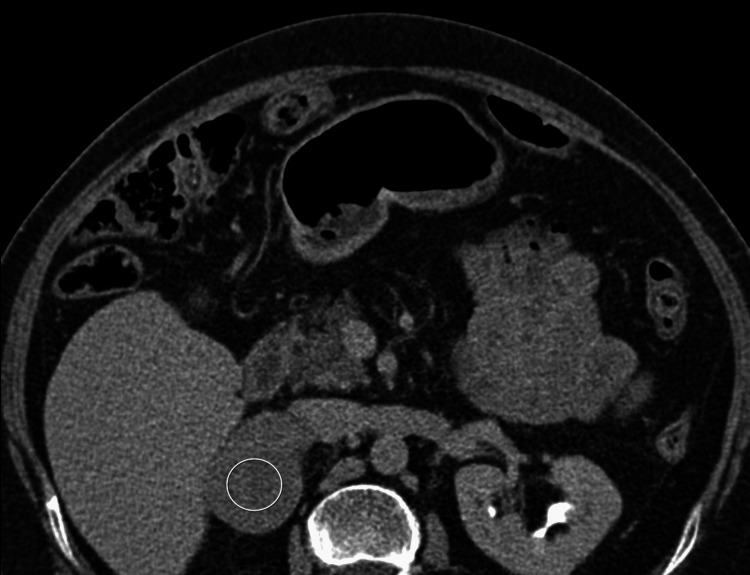
Computed tomography (CT) scan of the abdomen demonstrating incidentally noted adrenal mass. *White circle*: Large irregular right-sided adrenal mass with foci of heterogenous internal enhancement noted

A 75-year-old female was evaluated by endocrinology for an incidentally discovered adrenal mass. A week prior, she was hospitalized for chest pain. A CT angiogram to exclude aortic dissection revealed a large right adrenal lesion with foci of heterogeneous internal enhancement measuring 5.0 cm x 3.7 cm (Figure 1).

She was initially asymptomatic, and denied constitutional symptoms such as fatigue or unexplained loss of weight. However, she had a history of hypertension and anxiety, which raised concern for a pheochromocytoma. She otherwise denied unexplained bruising, palpitations, muscle aches, tremors, and heat/cold intolerance.

Aside from hypertension and anxiety, she had a history of type II diabetes mellitus managed on metformin alone. Her family history was remarkable for a brother who also had a left adrenal lesion which was found to be a non-functional adenoma following adrenalectomy.

Her vitals were normal except for a blood pressure of 150/90. Examination showed a well-nourished female with no obvious Cushingoid features, such as increased dorsocervical fat pad, axillary or abdominal striations, or unexplained extremity bruising. Cardiac and respiratory exams were within normal limits, and no lymphadenopathy was appreciated.

She was scheduled for further workup of her adrenal incidentaloma and was found to have an elevated serum cortisol level. An overnight low-dose dexamethasone suppression test was non-suppressed, and adrenocorticotropic hormone (ACTH) level was found to be low (Table 1). These findings confirmed autonomous adrenal production of corticosteroids, and she was referred to surgery for adrenalectomy.

**Table 1:** Investigations performed in the workup of the patient's incidentaloma. Repeat values for select investigations are presented a year later after she presented with metastatic disease. ACTH: adrenocorticotropic hormone

Investigation (units)	Value (initial)	Value (repeat)	Reference range
24-hour urinary epinephrine (mcg/24hr)	<1.4	-	<21
24-hour urinary norepinephrine (mcg/24hr)	28	-	15-80
24-hour urinary metanephrines (mcg/24hr)	<29	-	30-180
24-hour urinary normetanephrines (mcg/24hr)	211	-	148-560
Plasma renin activity (ng/mL/hr)	0.2	-	0.2-1.6
Serum aldosterone (ng/dL)	4.1	-	2-9
Serum cortisol (ug/dL)	22.2	54.1	2.7-10.5 (for 6-8PM)
24-hour urinary cortisol (mcg/day)	22.9	1347	<45
ACTH level (pg/mL)	3.2	-	7.2-63.3

**Figure 2:**
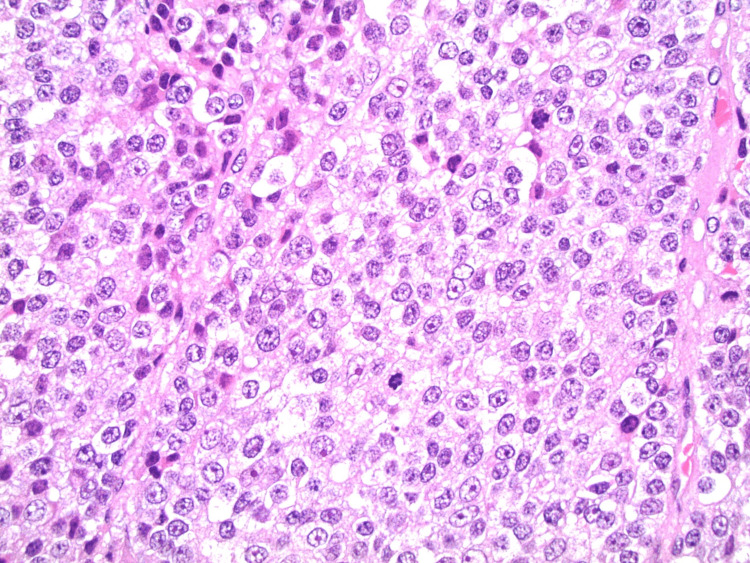
Hematoxylin & eosin stain of a section of tissue from pathologic biopsy under high power microscopy. Noted are the increased number of mitotic figures, increased nuclear:cytoplasmic ratio, and abnormal mitotic figures typical for a high-grade malignancy,

She successfully underwent surgery without complications. A surgical pathology report showed a high-grade adrenocortical carcinoma with positive surgical margins. Small vessel lymphovascular invasion was noted, but regional lymph nodes could not be assessed. The primary tumor was staged T2, with a mitotic rate of 22/50 high power fields that marked it as high grade histologically (Figure 2). 

She was subsequently referred to oncology for further evaluation, and proceeded with external beam radiation therapy for a total dose of 4500 cGy over 25 fractions, followed by adjuvant therapy with four cycles of carboplatin and etoposide. Dose reduction was needed after cycle two for worsening fatigue and neuropathy, but she otherwise tolerated the treatments well.

Nearly a year later, a regular surveillance CT demonstrated multiple sub-centimeter pulmonary nodules with patchy ground-glass abnormalities concerning for metastatic disease. In view of her disease progression, she started pembrolizumab immunotherapy.

**Figure 3:**
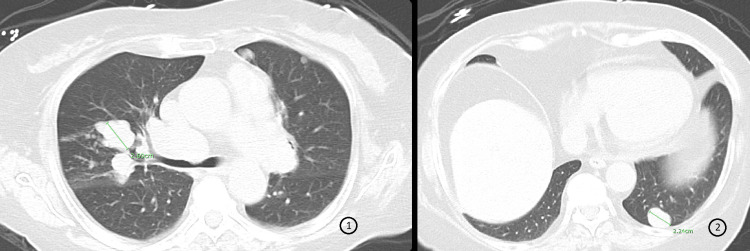
CT of the chest demonstrating multiple nodules in the lungs consistent with metastatic disease progression. *Green lines*: Identified lung parenchymal nodules measuring 2.60 cm (panel 1) and 2.24 cm (panel 2) in greatest diameter.

Repeat imaging, in the setting of worsening fatigue and anorexia, confirmed enlargement of her multiple lung nodules with a new soft tissue mediastinal mass also being found (Figure 3). She developed worsening lower extremity edema and required hospitalizations for recurrent hypokalemia with hypertension. Endocrinologic evaluation revealed grossly elevated 24-hour urinary free cortisol and elevated serum cortisol levels consistent with severe Cushing’s syndrome, and she was started on high-dose ketoconazole.

Despite six months of immunotherapy, repeat imaging showed substantial increase in size of both her multiple bilateral lung nodules. Extensive mediastinal and hilar adenopathy was also noted. Her treatment regimen was switched once more to mitotane 1g twice daily. She also had multiple subsequent hospitalizations for severe hypokalemia complicated by atrial fibrillation with rapid ventricular response.

She continued to clinically deteriorate, with increasing shortness of breath, fatigue, and chest pain. A goals of care discussion was held in view of her aggressive disease course and multiple lines of failed therapy. She was then transitioned to hospice care, and her mitotane was stopped.

## Discussion

Although overall adrenal tumors are common in the population, affecting about 3-10% of people, most of these are benign. ACC on the other hand is rare, and approximately 40-60% of ACCs are found to be functional tumors that produce hormones. Fifty to 80% of these functional ACCs secrete cortisol [4]. A surprising percentage of these may even be picked up incidentally, with one multicentric and retrospective evaluation of 1096 cases demonstrating that 12% of adrenal incidentalomas are ACCs [2]. Despite improved detection rates, however, this has not translated to earlier detection and treatment of ACC [5].

The first proposed TNM staging classification scheme for ACC in 2003 by the International Union Against Cancer (UICC) had notable shortcomings, including similar outcomes for both stage II and III disease [6]. A study of 492 patients in a German ACC registry found that disease-specific survival (DSS) did not significantly differ between stage II and stage III ACC (hazard ratio, 1.38; 95% confidence interval, 0.89-2.16) and furthermore, patients who had stage IV ACC without distant metastases had an improved DSS compared with patients who had metastatic disease (P = .004) [7]. The American Joint Committee of Cancer (AJCC), and the European Network for the Study of Adrenal Tumors (ENSAT) consequently developed revised staging systems that better reflect patient prognosis.

The most important predictors of survival in patients with ACC are tumor grade, tumor stage, and surgical treatment. For patients after surgical resection, the administration of adjunctive therapy is guided by the risk of recurrence. Despite early-stage resection, disease recurrence rates in ACC are very high. Besides the EDP-M regimen, no others have been successfully evaluated in large, randomized trials [4]. Whenever possible, it is still recommended that patients be referred to a clinical trial on an individual basis.

The ADJUVO clinical trial consisted of 91 low-recurrence-risk ACC patients who were randomly assigned to either observation or adjuvant mitotane therapy after surgical resection. Low recurrence risk is defined as Ki67<10%, stage I-III according to ENSAT classification, and microscopically complete resection. Adjuvant mitotane treatment failed to demonstrate statistically significant differences in disease-free survival, recurrence-free survival and overall survival between these patient groups [8]. Our case seems to suggest that even limited-stage disease may need to be managed aggressively not just with primary surgery, but also adjuvant chemoradiotherapy, especially with a high histologic grade. 

PD-1 blockade in adrenocortical carcinoma was evaluated in a phase II study of 39 participants, with a progression-free survival of 2.1 months independent of mismatch repair deficiency status being reported [9]. Despite switching to pembrolizumab in our patient, disease progression continued unabated, calling into question the clinical benefit of PD-1 blockade in ACC. 

A small study on the use of metyrapone with EDP-M in three advanced ACC patients with Cushing’s syndrome displayed a good safety profile with minor drug-drug interactions and appears to be a good option in combination with mitotane and other cytotoxic chemotherapies [10]. Ketoconazole is often less effective than metyrapone and requires regular monitoring of liver function tests, although it also inhibits androgen production.

## Conclusions

This case demonstrates the unfortunate prognosis of many patients with ACC. Although patients may present with classical symptoms of hypercortisolism or hyperandrogenism, many patients do not present with symptoms until the disease has advanced. Surgery may be employed with curative intent, although the evidence for adjuvant radiotherapy is mixed. The management for patients with ACC continues to remain a challenge due to the lack of evidence for optimal therapeutic management. In view of the aggressive nature of ACC, patients with high-grade histology despite limited-stage disease require adjuvant chemoradiation in addition to primary surgery to maximize the chances of progression-free survival. Also, although the use of PD-1 blockade has revolutionized cancer care in several other tumor types, evidence of clear benefit in ACC is lacking, as our case demonstrates.
